# Maternal region of birth and stillbirth trends in Victoria, Australia, 2012–2019: a cohort study

**DOI:** 10.1136/bmjph-2025-003004

**Published:** 2025-10-21

**Authors:** Natalie Holowko, Natalie Y Liu, Sharon Weerasingha, Roshan Selvaratnam, Mary-Ann Davey, Tanya Farrell, Miranda Davies-Tuck

**Affiliations:** 1 The Ritchie Centre, Hudson Institute of Medical Research, Clayton, Victoria, Australia; 2 Department of Obstetrics and Gynaecology, Monash University, Melbourne, Victoria, Australia; 3 Safer Care Victoria, Department of Health and Human Services, Victorian Government, Melbourne, Victoria, Australia

**Keywords:** Epidemiology, Public Health, Public Health Practice, Sociodemographic Factors, Community Health

## Abstract

**Introduction:**

Given the increasing attention to reduce stillbirth rates in migrant women and variable clinical guidance, we quantified contemporary trends in stillbirth based on maternal region of birth.

**Methods:**

Population-based study of singleton births ≥20 weeks of gestation between 2012 and 2019 from the Victorian Perinatal Data Collection (N=571 998). We investigated the association between maternal self-reported region of birth and stillbirth overall using logistic regression, and gestation and timing of stillbirth using multinomial regression. Trends in stillbirth overall, iatrogenic births, admission to a neonatal intensive care unit or special care nursery, neonatal death and cause of stillbirth by region of birth were also investigated.

**Results:**

Rates of stillbirth were significantly higher in women born in Africa (adjusted OR (aOR) 1.76: 1.47 to 2.12) and South Asia (aOR 1.26: 1.11 to 1.43) when compared with locally born non-Indigenous women. For women born in Africa, this was irrespective of gestation, while for women born in South Asia, this was specific to preterm stillbirths. Overall, we observed a decreasing trend in stillbirth among women born in Australia, while the stillbirth rate remained consistently higher and did not significantly decrease in women born in Africa, Oceania, New Zealand and South Asia. Over the same period, iatrogenic births significantly increased. Causes of stillbirth also differed by maternal region of birth.

**Conclusions:**

While we observed some improvements in stillbirth rates and increasing rates of iatrogenic birth, maternal region of birth remains an independent risk factor for stillbirth requiring targeted approaches.

**WHAT IS ALREADY KNOWN ON THIS TOPIC**

- Reducing the higher stillbirth rate among migrant women is a priority.

**WHAT THIS STUDY ADDS**

- Maternal region of birth remains an independent risk factor for stillbirth.
- Despite increasing trends in iatrogenic deliveries, we found persisting disparities in rates of stillbirth among women born in Africa, South Asia and Oceania.
- No significant difference in term stillbirth rates was found in women born in South Asia.

**HOW THIS STUDY MIGHT AFFECT RESEARCH, PRACTICE OR POLICY**

- Further investigation of early pre-conception factors and intra-pregnancy interventions is needed to achieve meaningful reductions in stillbirth rates among women born outside of Australia.
- A targeted approach is required to reduce inequalities in stillbirth.

## Background

 Despite a global decline, approximately 1.9 million late gestation stillbirths (≥28 weeks) occur annually.^1^ In 2019, the stillbirth rate ranged widely between 1.4 and 32.2 stillbirths per 1000 births,^2^ with the highest rates occurring in sub-Saharan Africa, followed by Southern Asia, North Africa and West Asia.^3^ These regions are the largest and growing sources of migration to Australia^4 5^ and other high-income countries,^6^ with high rates of stillbirth among women from these regions persisting even after migration.

Reducing stillbirth rates in migrant women is a priority,^7^ however evidence to achieve this is lacking. The drivers of elevated migrant stillbirth rates are multifactorial, reflecting complex biological, social and structural intersectionality.^8^,^10^ While several strategies have been explored—including group antenatal care, fetal monitoring and cultural training for midwives^11^—evidence is predominantly from small observational or qualitative studies and administrative data,^12 13^ limiting their routine clinical uptake. Consequently, these interventions are inconsistently supported across clinical guidelines and position statements,^14^,^18^ sparking ongoing debate^19^,^21^ and contributing to practice variation and potential harm.^22^

Higher stillbirth rates among women from South Asia, Africa and the Middle East have been documented in Victoria, Australia (2000–2011),^4^ and in other Australian jurisdictions.^5^ Investigating stillbirth trends after this period is important, given the introduction of key initiatives—such as fetal growth reporting,^23^ induction of labour^24^ and the Bridging the Gap^25^ programme—without which we could have expected an increase in stillbirths due to a higher stillbirth risk profile among mothers (older maternal age, higher body mass index (BMI) and increased numbers of mothers born in Oceania, Asia and Africa).^26^ Although the Safer Baby Bundle^27^ recommends increased surveillance of high-risk groups (women with a high BMI or who smoke) and a risk assessment for all women from 34 gestational weeks, clinical guidance for migrant women remains underexplored. A recent systematic review of global antenatal guidelines identified three migrant-specific interventions—fetal monitoring, group antenatal care and cultural training for midwives—that were associated with a reduced risk of stillbirth and increased rates of induction.^11^ With rising migration, limited clinical guidance and a focus to reduce stillbirths in migrant women, we investigated contemporary trends (2012–2019) in stillbirth rates by maternal region of birth, including timing and causes, as well as trends in iatrogenic births and potential neonatal harms, to address gaps in clinical guidance.

## Methods

### Study design and setting

This retrospective cohort study used anonymised data from the population-based Victorian Perinatal Data Collection (VPDC) database between 2012 and 2019. The VPDC is a legislated database including routinely collected and detailed maternal, pregnancy and birth information for all births in Victoria at 20 or more weeks of gestation (or with a birth weight of at least 400 g if gestation is not known). Detailed information of all stillbirths and neonatal deaths (NND) is reviewed by the independent Consultative Council on Obstetric and Paediatric Mortality and Morbidity and classified according to the Perinatal Society of Australia and New Zealand (PSANZ) Perinatal Death Classification system (PDC) version 3.2.^21^ Validation of the VPDC shows >95% accuracy for 78 of 111 variables and >90% accuracy for another 18.^28^

### Participants

We included singleton births ≥20 weeks of gestation and excluded pregnancies for Aboriginal and/or Torres Strait Islander women, due to existing disparities in stillbirth rates (compared with non-Indigenous women born in Australia) that warrant thorough investigation led by Indigenous researchers, as per Australian guidelines. We excluded births with a known (or inadequately defined) congenital anomaly or that ended in a termination (31 083 births), with our final sample including 571 998 births (90% of births reported to the VPDC). For our secondary analysis investigating the causes of stillbirth by region, we included congenital anomalies and terminations of pregnancy, in alignment with and comparable to classifications of causes of death globally.

### Exposure

Maternal self-reported country of birth was classified into regions of birth established by the United Nations (UN)—Australia, New Zealand, Oceania, Africa, Middle East (including Iran, Iraq, Jordan, Turkey, Yemen, Syria, Cyprus), South Asia (including India, Pakistan, Sri Lanka, Afghanistan, Bangladesh), South-East and East Asia (including Vietnam, Malaysia, Indonesia, China, Japan), Europe or ‘other’. While these UN groupings are identified as simplified policy-relevant regions of birth, heterogeneity exists within these regions. Non-Indigenous ‘Australia born’ was used as the reference category in all analyses.

### Outcomes

The primary outcome was stillbirth, defined as a fetal death at ≥20 weeks of gestation (or birth weight ≥400 g where gestation was unknown).

Secondary outcomes include gestation of stillbirth (20^+0^ to 36^+6^ weeks, or ≥37^+0^ onwards); timing of stillbirth (antepartum, intrapartum); iatrogenic birth (defined as having an induction of labour, a failed induction, no labour or a pre-labour caesarean section); admission to neonatal intensive care unit (NICU) or special care nursery (SCN); NND (defined as the death of a live-born infant, less than 28 days after birth, of any gestation or, if gestation is unknown, weighing at least 400 g).

Cause of stillbirth was categorised according to the PSANZ PDC system version 3.2.^21^ Given a restructuring of the PDC system in 2017, all deaths prior to this were harmonised with the new classification, which separates the previous category of ‘specific perinatal conditions’ into either ‘complications of multiple pregnancy’ or ‘specific perinatal conditions’, and renames ‘fetal growth restriction’ to ‘placental dysfunction or causative placental pathology’.

### Covariates

The following variables were included for both descriptive statistics and also modelling of our outcomes: maternal age (<25, 25–29, 30–34, 35–39 and ≥40 years); parity (primiparous, multiparous); weight status—BMI group (kg/m^2^) (<18.5, 18.5–24.9, 25–29.9 and ≥30); assisted reproductive technology (ART); social disadvantage (indicator of relative socioeconomic disadvantage (IRSD), quintiles derived by the VPDC modelled a continuous variable); year of birth of child; smoking status (non-smoker, quit smoking <20 weeks gestation, smoker); and timing of first antenatal care appointment (<12 weeks, 12–17 weeks, ≥18 weeks, did not attend).

The following variables (derived using ICD-10-AM codes) were used only for descriptive statistics: previous caesarean section; previous stillbirth; pre-existing maternal conditions (diabetes, hypertension, thyroid); maternal conditions during pregnancy (gestational diabetes mellitus, hypertension); sex; and birth weight below 3rd centile.^29^

### Statistical analyses

Maternal, pregnancy and birth characteristics of the study population were presented by maternal region of birth and compared using t-tests and χ^2^ tests as appropriate. Cell sizes <5 were reported as ‘<5’. For both stillbirth and iatrogenic births, the rate per 1000 births was calculated for each maternal region of birth per year using the fetuses-at-risk approach,^30^ which estimates gestational-age specific risk by calculating the number of stillbirths occurring in that week divided by the total number of births (live and stillbirths) from that week onwards. By region of birth and year, we plotted the rates of stillbirth, iatrogenic births, NICU or SCN admissions and NND, and causes of stillbirth, testing for trends using a Mann-Kendall non-parametric test with a two-sided p value.

For all models, we present a minimally (including birth year, for possible cohort effects) and fully adjusted model (model 1) including covariates identified a priori (maternal age, primiparity, BMI, IRSD, ART, iatrogenic birth, smoking status and antenatal care attendance).^4 5^

The association between maternal region of birth and stillbirth (compared with live birth) was estimated using logistic regression. In the minimally adjusted model, we separately included an interaction term between region of birth and iatrogenic birth, maternal age and giving birth <37 weeks. Sensitivity analysis was performed, restricting to births ≥24 weeks. We used multinomial logistic regression to model the association between maternal region of birth and the odds of (a) gestation of stillbirth (20^+0^ to 36^+6^ weeks or ≥37 weeks, compared with a live birth); and (b) timing of stillbirth (antepartum or intrapartum stillbirth, compared with a live birth).

Data management and statistical analyses were conducted using SAS V.9.4 (SAS Institute, Cary, North Carolina, USA), R V.4.4.1 (R Foundation for Statistical Computing, Vienna, Austria) and R Studio V.2024.04.2 (http://www.rstudio.com).

### Patient and public involvement

Patients and/or the public were not involved in the design, conduct, reporting or dissemination of this research.

## Results

38% of women (n=217 021) were not born in Australia. Overall, 12% were born in South-East and East Asia, 11% in South Asia, 3% in Africa and 2% in the Middle East. Characteristics of women are presented overall (online supplemental table 1) and stratified by region of birth (table 1 and online supplemental table 1). Women born in New Zealand, Oceania, the Middle East and South Asia were more likely to be younger, while those born in Australia and Europe were more likely to be ≥35 years of age. Primiparity was more common among women born in South-East and East Asia and South Asia.

**Table 1:** Maternal and early pregnancy characteristics and pre-existing conditions by maternal region of birth* (N=571 988)

Characteristics	Australia (N=354 977, 62%)	New Zealand (N=12 573, 2.2%)	Oceania (N=3850, 0.6%)	South-East and East Asia (N=67 271, 11.8%)	South Asia (N=64 299, 11.2%)	Europe (N=26 575, 4.6%)	Africa (N=18 179, 3.2%)	Middle East (N=12 113, 2.1%)
Maternal age (years)								
<25 years	45 253 (12.7)	2141 (17.0)	639 (16.6)	3945 (5.9)	4718 (7.3)	1231 (4.6)	2338 (12.9)	2129 (17.6)
25–29 years	90 439 (25.5)	3155 (25.1)	984 (25.6)	16 680 (24.8)	21 248 (33.0)	4638 (17.5)	4956 (27.3)	3701 (30.6)
30–34 years	128 843 (36.3)	3918 (31.2)	1214 (31.5)	27 683 (41.2)	27 538 (42.8)	10 425 (39.2)	6146 (33.8)	3565 (29.4)
35–39 years	73 147 (20.6)	2738 (21.8)	753 (19.6)	15 527 (23.1)	9500 (14.8)	8252 (31.1)	3697 (20.3)	2122 (17.5)
≥40 years	17 295 (4.9)	621 (4.9)	260 (6.8)	3436 (5.1)	1295 (2.0)	2029 (7.6)	1042 (5.7)	596 (4.9)
Parity								
0	155 128 (43.7)	4918 (39.1)	1128 (29.3)	32 509 (48.3)	31 474 (48.9)	12 286 (46.2)	5320 (29.3)	3567 (29.4)
1	127 064 (35.8)	3888 (30.9)	1054 (27.4)	25 254 (37.5)	24 986 (38.9)	9991 (37.6)	5150 (28.3)	3696 (30.5)
2	50 408 (14.2)	1842 (14.7)	621 (16.1)	7058 (10.5)	5457 (8.5)	3195 (12.0)	3115 (17.1)	2580 (21.3)
≥3	22 365 (6.3)	1922 (15.3)	1047 (27.2)	2447 (3.6)	2378 (3.7)	1100 (4.1)	4591 (25.3)	2267 (18.7)
Missing	12 (0.0)	<5	0	<5	<5	<5	<5	<5
Body mass index (WHO BMI, kg/m^2^)								
<18.5 underweight	7788 (2.2)	119 (0.9)	64 (1.7)	4869 (7.2)	1807 (2.8)	634 (2.4)	556 (3.1)	202 (1.7)
18.5–24.9 normal weight	157 541 (44.4)	4189 (33.3)	832 (21.6)	43 957 (65.3)	29 836 (46.4)	14 670 (55.2)	7294 (40.1)	4649 (38.4)
25.0–29.9 overweight	95 127 (26.8)	3184 (25.3)	911 (23.7)	11 544 (17.2)	21 340 (33.2)	6441 (24.2)	5142 (28.3)	3720 (30.7)
≥30 obese	79 749 (22.5)	4393 (34.9)	1914 (49.7)	3097 (4.6)	8601 (13.4)	3218 (12.1)	3474 (19.1)	2584 (21.3)
Missing	14 772 (4.2)	688 (5.5)	129 (3.4)	3804 (5.7)	2715 (4.2)	1612 (6.1)	1713 (9.4)	958 (7.9)
IRSD (indicator of relative social deprivation)								
1	57 454 (16.9)	2442 (20.0)	1266 (33.9)	15 413 (23.6)	16 590 (26.7)	2970 (11.6)	6406 (36.4)	4934 (42.0)
2	69 441 (20.5)	2624 (21.5)	959 (25.7)	11 310 (17.3)	14 028 (22.5)	4119 (16.1)	3287 (18.7)	2593 (22.1)
3	71 044 (20.9)	2472 (20.3)	672 (18.0)	11 857 (18.2)	13 144 (21.1)	4873 (19.0)	2754 (15.6)	1814 (15.4)
4	69 735 (20.6)	2359 (19.3)	512 (13.7)	13 332 (20.4)	11 219 (18.0)	5934 (23.2)	2677 (15.2)	1418 (12.1)
5	71 643 (21.1)	2296 (18.8)	324 (8.7)	13 282 (20.4)	7261 (11.7)	7685 (30.0)	2489 (14.1)	985 (8.4)
Assisted reproductive technology used	14 852 (4.2)	402 (3.2)	66 (1.7)	1768 (2.6)	1528 (2.4)	1330 (5.0)	338 (1.9)	279 (2.3)
Admission status								
Public	248 141 (69.9)	10 664 (84.8)	3460 (89.9)	47 516 (70.6)	55 173 (85.8)	19 076 (71.8)	15 852 (87.2)	10 693 (88.3)
Private	97 172 (27.4)	1693 (13.5)	354 (9.2)	18 425 (27.4)	7920 (12.3)	6889 (25.9)	2092 (11.5)	1291 (10.7)
Missing	9664 (2.7)	216 (1.7)	36 (0.9)	1330 (2.0)	1206 (1.9)	610 (2.3)	235 (1.3)	129 (1.1)
Previous stillbirth	4347 (1.2)	189 (1.5)	65 (1.7)	592 (0.9)	907 (1.4)	274 (1.0)	454 (2.5)	281 (2.3)
Previous caesarean section	59 809 (16.8)	1839 (14.6)	644 (16.7)	9984 (14.8)	12 318 (19.2)	4056 (15.3)	4114 (22.6)	2329 (19.2)
Pre-existing diabetes mellitus	2708 (0.8)	126 (1.0)	78 (2.0)	550 (0.8)	840 (1.3)	144 (0.5)	187 (1.0)	96 (0.8)
Pre-existing hypertension	4256 (1.2)	156 (1.2)	34 (0.9)	430 (0.6)	458 (0.7)	185 (0.7)	184 (1.0)	84 (0.7)
Gestational age at first antenatal visit								
<12 weeks	180 130 (50.7)	4453 (35.4)	1115 (29.0)	29 865 (44.4)	24 861 (38.7)	12 094 (45.5)	5569 (30.6)	4282 (35.4)
12–17 weeks	112 215 (31.6)	4153 (33.0)	1169 (30.4)	23 423 (34.8)	25 286 (39.3)	9414 (35.4)	6387 (35.1)	4357 (36.0)
≥18 weeks	59 594 (16.8)	3797 (30.2)	1498 (38.9)	13 516 (20.1)	13 850 (21.5)	4891 (18.4)	6037 (33.2)	3385 (27.9)
None or inadequately defined	3031 (0.9)	170 (1.4)	68 (1.8)	466 (0.7)	302 (0.5)	176 (0.7)	186 (1.0)	89 (0.7)
Missing	7 (0.0)	0	0	<5	0	0	0	0
Smoking status at 20 weeks gestation								
Non-smoker	291 173 (82.0)	10 001 (79.5)	3310 (86.0)	64 622 (96.1)	62 832 (97.7)	23 979 (90.2)	17 318 (95.3)	11 337 (93.6)
Quit smoking <20 weeks	7103 (2.0)	337 (2.7)	72 (1.9)	461 (0.7)	139 (0.2)	527 (2.0)	144 (0.8)	176 (1.5)
Smoker	52 214 (14.7)	2079 (16.5)	440 (11.4)	1460 (2.2)	1164 (1.8)	1815 (6.8)	633 (3.5)	522 (4.3)
Missing	4487 (1.3)	156 (1.2)	28 (0.7)	728 (1.1)	164 (0.3)	254 (1.0)	84 (0.5)	78 (0.6)

* Characteristics overall (N=571 998) and for women born in ‘other’ regions (N=12 161, 2.1%) are shown in online supplemental table 1.

Smoking during pregnancy was more prevalent among women born in Australia, New Zealand and Oceania (11%–15%), and below 7% for all other regions, and was lowest for South Asia and South-East and East Asia born. BMI≥25 was highest for women born in Oceania (73%), New Zealand (60%) and Australia (49%), and lowest for women born in South-East and East Asia, South Asia and Europe (10%–18%). Social disadvantage (IRSD Q1-Q2) affected >50% of women born in the Middle East, Oceania, Africa and South Asia, compared with 37% of Australia-born women. Pre-existing diabetes was more common among women born in Oceania and South Asia than in Australia, Europe or ‘other’ regions, while pre-existing hypertension was highest among women born in Australia and New Zealand and lowest in South-East and East Asia born women.

Half of women born in Australia engaged in antenatal care <12 weeks gestation, compared with less than one-third of women born in Oceania and Africa. The highest proportions of women not attending antenatal care were born in Oceania and New Zealand (online supplemental table 2).

Gestational diabetes affected 11% of women and was highest among women born in South Asia, Oceania, South-East and East Asia, and the Middle East (online supplemental table 2). Gestational hypertension and preeclampsia were most common among women born in Oceania. While ~50% had a spontaneous onset of labour, the proportion of iatrogenic births was higher among women born in South Asia (57% vs overall average 52%).

Overall, 6.3% of women had an infant born between 20^+0^ and 36^+6^ weeks of gestation, and this was slightly higher among women born in Oceania (7.1%) (online supplemental table 2). While 2% of infants had birth weight <3rd centile, this doubled among women born in South Asia. Overall, 1.2% of infants were admitted to NICU, and this was slightly higher if the mother was born in Africa (1.9%) or Oceania (1.5%). The proportion of NND (overall 0.1%) was highest for mothers born in Oceania (0.3%), Africa (0.2%) and South Asia (0.2%) (overall rate per 1000 births was 1.16 among women born in Australia, 2.86 among women born in Oceania, 1.87 among women born in Africa and 1.63 among women born in South Asia—results not shown).

### Region of birth and stillbirth

The overall stillbirth rate between 2012 and 2019 was 4.1 per 1000 births among women born in Australia (table 2); this was almost doubled for women born in Africa (7.8 per 1000), 60% higher for Oceania (6.5 per 1000) and ~25% higher for the Middle East (5.2 per 1000) and South Asia (5.1 per 1000).

**Table 2:** Association between region of birth and stillbirth in Victoria, 2012–2019—overall and stratified by gestation at birth (20^+0^–36^+6^ or ≥37 weeks gestation)

	Stillbirth rate per 1000 births	Minimal model		Model 1	
		OR	95% CI	OR	95% CI
Overall	4.3				
Region of birth*					
Australia	4.1	1.00		1.00	
New Zealand	4.9	1.18	0.91 to 1.53	1.18	0.91 to 1.54
Oceania	6.5	1.62	1.09 to 2.41	1.47	0.98 to 2.22
South-East and East Asia	3.2	0.79	0.69 to 0.92	0.87	0.74 to 1.01
South Asia	5.1	1.27	1.13 to 1.43	1.26	1.11 to 1.43
Europe	3.4	0.82	0.66 to 1.02	0.89	0.72 to 1.11
Africa	7.8	1.94	1.63 to 2.31	1.76	1.47 to 2.12
Middle East	5.1	1.13	0.87 to 1.47	1.20	0.93 to 1.56
Other	4.5	1.10	0.84 to 1.45	1.16	0.88 to 1.54
Stratified by gestation at birth†					
20^+0^ to 36^+6^ weeks gestation	3.3				
Australia	3.2	1.00		1.00	
New Zealand	3.4	1.06	0.78 to 1.45	1.07	0.78 to 1.46
Oceania	4.9	1.56	0.99 to 2.46	1.38	0.86 to 2.21
South-East and East Asia	2.5	0.77	0.66 to 0.92	0.83	0.70 to 0.99
South Asia	4.0	1.28	1.11 to 1.46	1.28	1.11 to 1.48
Europe	2.7	0.83	0.65 to 1.06	0.89	0.69 to 1.14
Africa	5.8	1.85	1.51 to 2.26	1.66	1.34 to 2.05
Middle East	3.9	1.26	0.94 to 1.68	1.17	0.87 to 1.57
Other	3.8	1.17	0.87 to 1.58	1.21	0.89 to 1.65
≥37 weeks gestation	0.9				
Australia	0.9	1.00		1.00	
New Zealand	1.5	1.60	0.98 to 2.61	1.63	0.98 to 2.70
Oceania	1.7	1.83	0.82 to 4.11	1.86	0.82 to 4.20
South-East and East Asia	0.8	0.86	0.64 to 1.17	0.99	0.72 to 1.35
South Asia	1.1	1.25	0.96 to 1.63	1.17	0.88 to 1.55
Europe	0.7	0.75	0.46 to 1.22	0.87	0.53 to 1.42
Africa	2.1	2.28	1.61 to 3.23	2.17	1.50 to 3.14
Middle East	1.2	1.37	0.79 to 2.34	1.34	0.78 to 2.31
Other	0.8	0.86	0.45 to 1.68	0.97	0.50 to 1.88

All births ≥20+0 weeks gestation (excluding multiple births, congenital anomalies, births to Aboriginal women and terminations of pregnancy). ORs (with 95% CIs) compared with having a live birth, estimated using logistic* and multinominal logistic† regression models (N=571 998).

Minimal model=adjusted for year of birth. Model 1=adjusted for maternal age at birth, primiparity (ref=yes), BMI (ref=‘healthy weight’ 18.5–24.9, IRSD quintiles (continuous), assisted reproductive technology used (ref=no), iatrogenic birth (ref=no), smoking status (ref=non-smoker), gestational age at first antenatal care visit (ref=<12 weeks).

* There was no significant interaction between region of birth and the following variables in an unadjusted model: year of birth (p=0.065), maternal age (p=0.073), iatrogenic birth (p=0.703) and birth <37 weeks gestation (p=0.667).

† Results from a multinomial logistic regression modelling the odds of stillbirth at 20+0–36+6 weeks gestation or stillbirth at ≥37 weeks gestation, compared with having a live birth.

**Figure 1:**
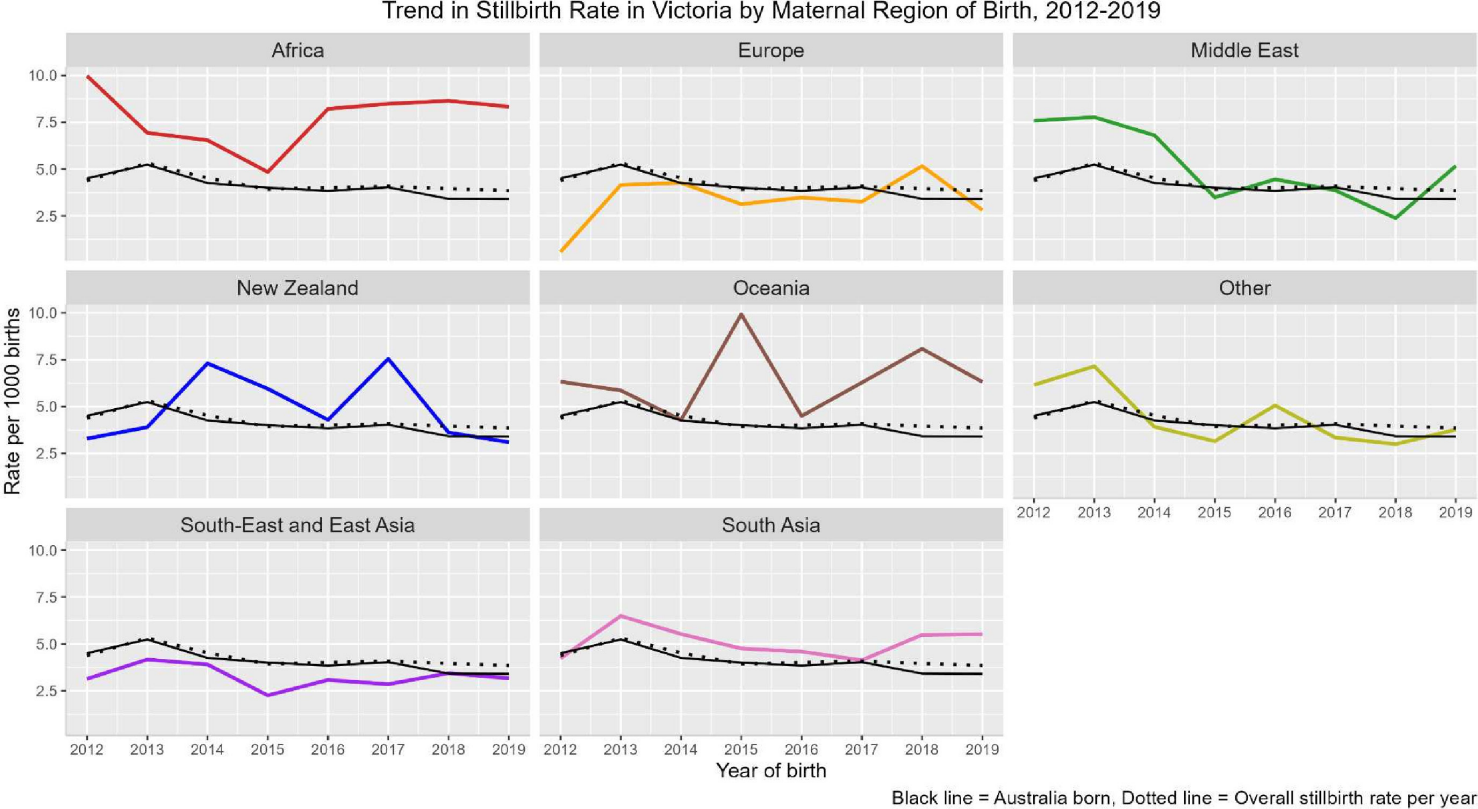
Trend in stillbirth rate panelled by maternal region of birth, compared to women born in Australia. Mann-Kendall two-sided p value was only significant for a downward trend in stillbirth among women born in Australia (p=0.00937).

While the overall stillbirth rate decreased 14% from 4.4 per 1000 births in 2012 to 3.9 per 1000 births in 2019 (figure 1), rates remained consistently higher among women who were born in Africa, Oceania, New Zealand and South Asia. While stillbirth rates fluctuated by region of birth, this was only significant for women born in Australia (negative trend, p=0.009).

After conditioning for covariates, women born in Africa (aOR 1.76: 95% CI 1.47 to 2.12) and South Asia (aOR 1.26: 95% CI 1.11 to 1.43) had increased odds of stillbirth (table 2). There was no significant association for women born in South-East and East Asia (aOR 0.87: 95% CI 0.74 to 1.01), Oceania (aOR 1.47: 95% CI 0.98 to 2.22), the Middle East (aOR 1.20: 95% CI 0.93 to 1.56) or New Zealand (aOR 1.18: 95% CI 0.91 to 1.54). No significant interaction was found between region of birth and maternal age (p=0.073), nor having an iatrogenic (p=0.703) or preterm birth (p=0.667), on the odds of stillbirth. Findings were similar in sensitivity analyses restricting to births ≥24 weeks of gestation (online supplemental table 3).

### Timing and gestation of stillbirth

Most stillbirths (79%) occurred between 20^+0^ to 36^+6^ weeks of gestation (online supplemental table 2). Regardless of gestation, stillbirth rates were highest among women born in Africa (20^+0^ to 36^+6^ weeks 5.8 per 1000, ≥37 weeks 2.1 per 1000), Oceania (20^+0^ to 36^+6^ weeks 4.9 per 1000, ≥37 weeks 1.7 per 1000), South Asia (20^+0^ to 36^+6^ weeks 4.0 per 1000, ≥37 weeks 1.1 per 1000), the Middle East (20^+0^ to 36^+6^ weeks gestation 3.9 per 1000, ≥37 weeks gestation 1.2 per 1000) and New Zealand (20^+0^ to 36^+6^ weeks 3.4 per 1000, ≥37 weeks 1.5 per 1000) (table 2). Women born in Africa (aOR 1.66: 95% CI 1.34 to 2.05) and South Asia (aOR 1.28: 95% CI 1.11 to 1.48) experienced higher odds of stillbirth at 20^+0^ to 36^+6^ weeks; however, only women born in Africa experienced higher rates at ≥37 weeks (aOR 2.17: 95% CI 1.50 to 3.14). Women born in South-East and East Asia had decreased odds of stillbirth at 20^+0^ to 36^+6^ weeks (aOR 0.83: 95% CI 0.70 to 0.99). All other regions of birth were not significantly associated with stillbirth, regardless of gestation.

The majority of stillbirths occurred antepartum (79%) (online supplemental table 2). After conditioning for potential confounders, there were increased odds of antepartum stillbirth for women born in Africa (aOR 1.76: 95% CI 1.43 to 2.16) and South Asia (aOR 1.23: 95% CI 1.07 to 1.42), compared with women born in Australia (online supplemental table 4). While intrapartum stillbirth rates among women born in Africa, Oceania and South Asia were higher, this was not statistically significant. Women born in South-East and East Asia had significantly reduced odds of intrapartum stillbirth (aOR 0.61: 95% CI 0.39 to 0.95).

### Iatrogenic births, stillbirth rates and perinatal harms

**Figure 2:**
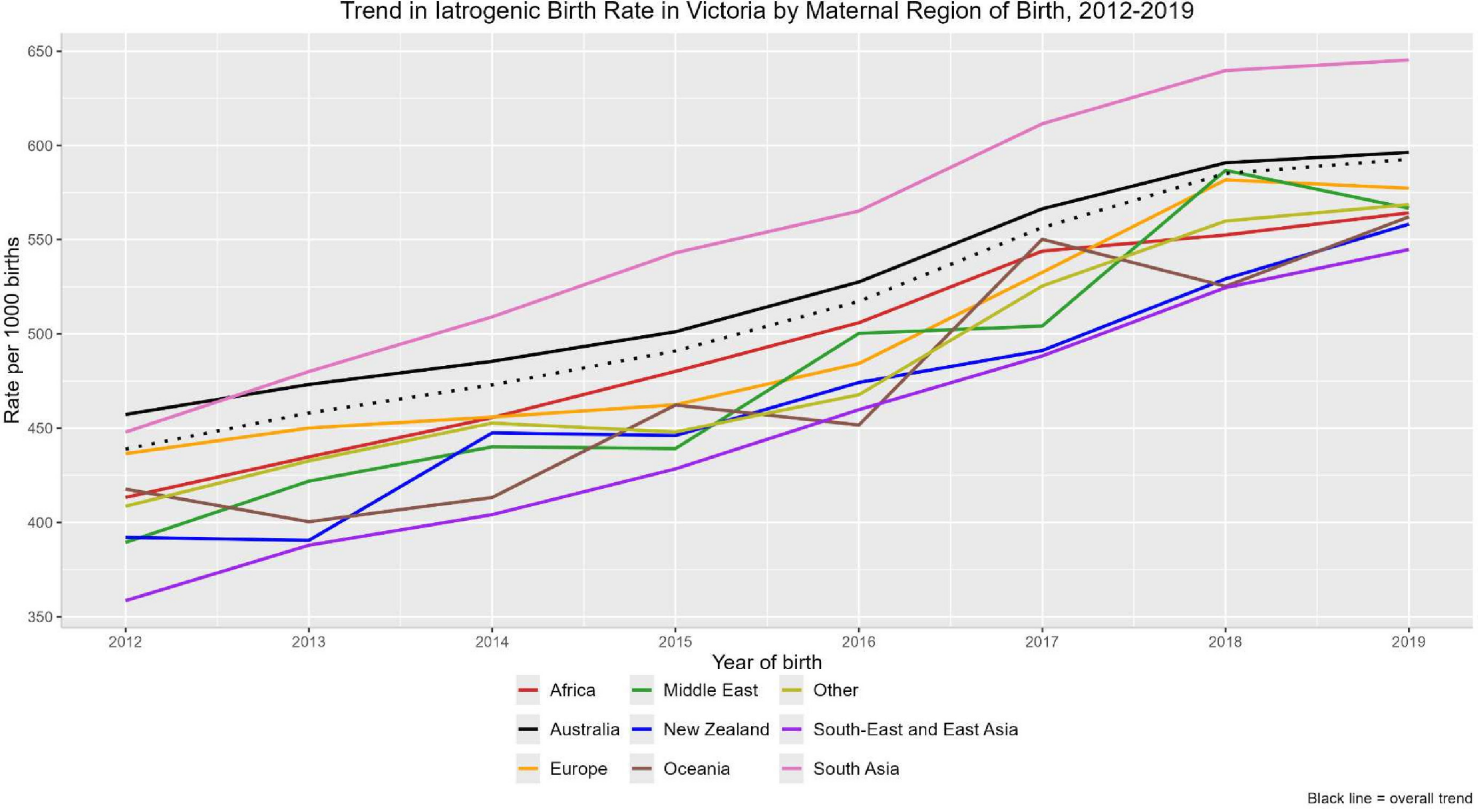
Trend in the rate of iatrogenic births between 2012 and 2019 by maternal region of birth. Mann-Kendall test for trend was positive and significant for all regions of birth: Africa (p=0.0008), Australia (p=0.0008), Europe (p=0.002), Middle East (p=0.0044), New Zealand (p=0.0044), Oceania (p=0.02), other (p=0.002), South-East and East Asia (p=0.0008), South Asia (p=0.0008).

**Figure 3:**
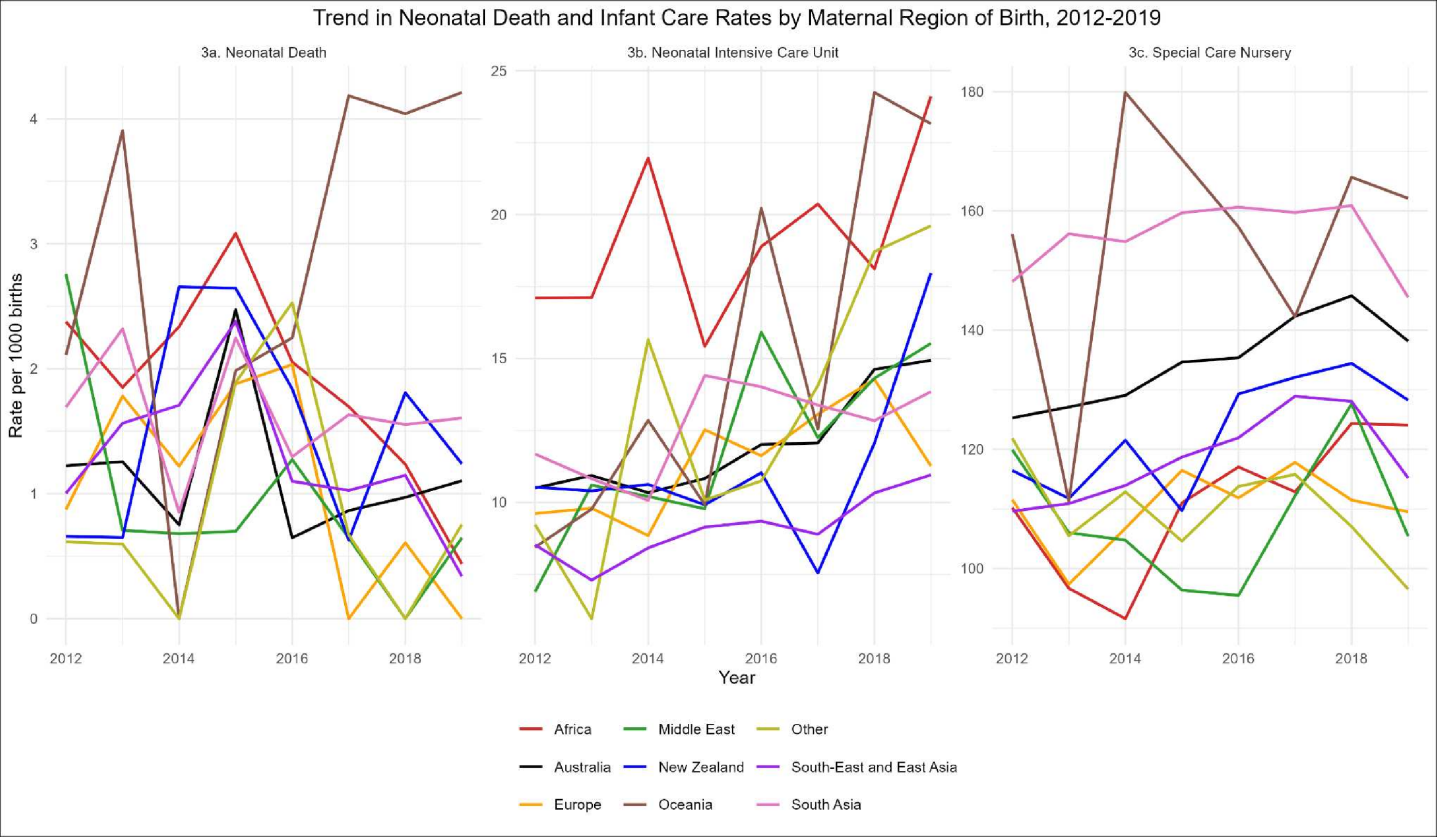
Trend in neonatal death and infant care (neonatal intensive care and special care nursery) rates by maternal region of birth, 2012–2019. Mann-Kendall test for trend significant for the following regions: neonatal death—decreasing trend: Africa (p=0.0354); neonatal intensive care unit—increasing trend: Australia (p=0.009), Oceania (p=0.0187), South-East and East Asia (p=0.0187), other (p=0.0187); special care nursery—increasing trend: Africa (p=0.0354), Australia (p=0.004), South-East and East Asia (p=0.0354).

The iatrogenic birth rate significantly increased by at least 30% between 2012 and 2019 for all regions of birth groups (figure 2), with an overall increase from approximately 45% to 59%. Increases were highest for women born in South-East and East Asia, the Middle East, South Asia and New Zealand. There was a significant decreasing trend over time in NND rate among women born in Africa (figure 3). Admissions to NICU also significantly increased among women born in Australia, Oceania, South-East and East Asia, and ‘other’ regions. Similar observations were seen for SCN admissions.

The main causes of stillbirth were maternal conditions, congenital anomalies and unexplained antepartum fetal death (online supplemental table 5 and figure 1). Stillbirths due to maternal conditions were approximately four times greater among women born in Oceania and three times higher among women born in New Zealand, compared with women born in Australia (online supplemental table 5). Women born in Africa had an almost threefold stillbirth rate due to spontaneous preterm labour and twofold due to antepartum haemorrhage, compared with women born in Australia.

## Discussion

While our population-based study demonstrated persisting disparities in rates of stillbirth for women born in Africa, South Asia and Oceania, notable improvements were also made. Specifically, we found no significant difference in term stillbirth rates in women born in South Asia. Rates of iatrogenic births, NICU and SCN admissions significantly increased, with no interaction between region of birth and iatrogenic birth on stillbirth odds. Both the causes and risk factors for stillbirth varied by region which, combined with a slower or absent reduction in stillbirth rates among women born abroad, highlights a need for targeted approaches to reduce the risk of stillbirth among migrant women.

Consistent with findings between 2000 and 2011, women born in South Asia and Africa continued to experience increased odds of stillbirth, compared with women born in Australia.^4^ For women born in Africa, the risk remained elevated irrespective of gestation, while for South Asia-born women, this was restricted to the preterm period. Women born in South-East and East Asia continued to have reduced odds of stillbirth,^4^ specifically preterm, antepartum and intrapartum. Given these findings, our study is the first to report no disparities in term stillbirth rates for women born in South Asia and no significant increased risk at any gestation for women born in the Middle East, unlike earlier analyses.^4^

While specific drivers for these reductions were beyond our scope, several key initiatives were implemented during this period, making analysis of trends beyond our prior research (births between 2000 and 2011)^4^ crucial. This includes public reporting of fetal growth restriction from 2012 and improved detection of severe small for gestational age (SGA),^23^ increased use of induction of labour^24^ and the Bridging the Gap^25^ programme. As confirmed elsewhere,^31^ women born in South Asia, Africa and Oceania were more likely to have SGA babies, therefore are most likely to have benefited from improvements in detection. From 2014 to 2016, the Bridging the Gap partnership introduced co-designed strategies to improve access to universal healthcare and maternity services for refugee-background families, including free culturally appropriate group pregnancy care initially for Karen families and then later for women of other backgrounds (South Sudanese, Assyrian Chaldean, Iraqi Muslim, Afghan and Vietnamese),^32^ with increased antenatal visits attended.^33^ A fetal surveillance programme for women born in South Asia was also implemented at Victoria’s largest health service, to detect fetal compromise from 39 weeks.^34^

Significant increases in iatrogenic births were observed, irrespective of region of birth, reflecting a shift in obstetric practice (increased surveillance, risk management and evolving guidelines around induction and caesarean delivery). Alongside women born in South-East and East Asia and the Middle East, the relative increase was greatest among women born in South Asia, a previously identified high-risk group.^4 35^ While iatrogenic birth at 41 weeks of gestation reduces stillbirth risk,^36^ the benefits are unclear for earlier births without clinical indication. A systematic review of seven trials (n=7598) found no association between induction from 39 weeks and reduced perinatal death,^37^ with one of the studies reporting a 54% higher risk of perinatal death for women born in Asia. The paradox of a universal increase in iatrogenic births, without a universal reduction in stillbirths and with increased NICU admissions, suggests that earlier induction may not prevent stillbirth and may introduce harms such as iatrogenic prematurity. This highlights the importance of risk-stratified approaches to timing of birth and induction of labour.

Reduced access and uptake of pregnancy care are established risk factors for stillbirth.^38^ Migrant and refugee women often face barriers to pregnancy care, including limited health system knowledge, language challenges, social isolation, limited support and a lack of culturally appropriate care.^39^ Consistent with other Australian studies,^40^ later antenatal care engagement was more common among women born in the Middle East, Oceania and South Asia, with women born in the Middle East and Africa most represented in care initiated at ≥18 weeks gestation. A systematic review has associated late antenatal care—common among immigrants and refugees outside universal healthcare—with poorer outcomes,^38^ highlighting the need to identify underlying factors preventing engagement. Despite hypotheses about reluctance towards medical interventions among women born in Africa,^41^ we found similar rates of elective (16% vs 18%) and emergency caesareans (17% vs 15%) among these women, compared with women born in Australia, and no major differences in intervention uptake once care was accessed.

While stillbirth rates were lower for all migrants than in their region of origin,^42^ increased rates among women born abroad remain an area for targeted improvements, including addressing cultural drivers/barriers (such as racism and language barriers, which are often inadequately measured) and tailoring maternity care to best care for women according to varying risks, in order to improve maternal and infant outcomes. A recent systematic review identified fetal monitoring, group antenatal care and cultural training for midwives as three initiatives that had a positive impact on reducing the risk of stillbirth among high-risk migrant women.^11^

While our study is strengthened by using validated, population-based register data including all pregnancies in Victoria, we were restricted by only having data on region of birth and not ethnicity. As previously identified,^4^ this may lead to misclassification of our exposure and possibly an overestimation or underestimation of our outcomes. According to the Australian Bureau of Statistics, the top five ancestries in 2021 were English (33%), Australian (29.9%), Irish (9.5%), Scottish (8.6%) and Chinese (5.5%).^43^ Further research is needed to quantify the extent of misclassification of ethnicity when using region of birth and whether duration of residence in Australia modifies the stillbirth risk for women born abroad. Residual confounding related to socioeconomic inequalities may remain, given that our data described area-level disadvantage and lacked individual factors contributing to social disadvantage. Further residual confounding could also remain due to other factors affecting women, such as stress, discrimination and nutrition. Although we had data on the gestational age at antenatal care initiation (a proxy for maternity care engagement), the total number of visits attended was not available, which would give a more comprehensive picture of care provided. Our findings may be limited to high-income countries that similarly have universal access to pregnancy care and comparable migration patterns.

## Conclusions

Maternal region of birth remains an independent risk factor for stillbirth, despite increasing trends in iatrogenic deliveries. Further investigation of early pre-conception factors and intra-pregnancy interventions is needed to achieve meaningful reductions in stillbirth rates among women born outside of Australia. We can expect little improvement without a targeted approach to reduce inequalities in stillbirth—our migrant-rich population deserves better.

## Supplementary material

10.1136/bmjph-2025-003004 online supplemental file 1

## Data Availability

Data may be obtained from a third party and are not publicly available.
