# Supplementary material for: Maternal region of birth and stillbirth trends in Victoria, Australia, 2012–2019: a cohort study
Source: BMJ Public Health. 2025 Oct 21;3(2):e003004. doi: 10.1136/bmjph-2025-003004 (PMC12551549; doi:10.1136/bmjph-2025-003004)
Supplement: online supplemental file 1 [file bmjph-3-2-s001.doc]

**Supplementary Table 1.** Maternal and early pregnancy characteristics and pre-existing conditions overall (N=571,988) and for women born in ‘Other’ regions (N=12,161)

| **Characteristics** | **Overall**  **(N = 571,998)** | **‘Other’ regions**  **(N = 12,161, 2.1%)** |
| --- | --- | --- |
| ***Maternal age (years)*** | |  |
| <25 years | 63,188 (11.0) | 794 (6.5) |
| 25-29 years | 148,306 (25.9) | 2,505 (20.6) |
| 30-34 years | 214,089 (37.4) | 4,757 (39.1) |
| 35-39 years | 119,044 (20.8) | 3,308 (27.2) |
| >= 40 years | 27,371 (4.8) | 797 (6.6) |
| ***Parity*** |  |  |
| 0 | 252,451 (44.1) | 6,121 (50.3) |
| 1 | 205,095 (35.9) | 4,012 (33.0) |
| 2 | 75,614 (13.2) | 1,338 (11.0) |
| >= 3 | 38,806 (6.8) | 689 (5.7) |
| Missing | 32 (0.0) | <5 |
| ***Body mass index (WHO BMI, kg/m^2^)*** | |  |
| <18.5 Underweight | 16,338 (2.9) | 299 (2.5) |
| 18.5-24.9 Normal weight | 269,207 (47.1) | 6,239 (51.3) |
| 25.0-29.9 Overweight | 150,491 (26.3) | 3,082 (25.3) |
| >=30 Obese | 108,854 (19.0) | 1,824 (15.0) |
| Missing | 27,108 (4.7) | 717 (5.9) |
| ***IRSD (Indicator of relative social deprivation)*** | |  |
| 1 | 109,266 (19.9) | 1,791 (15.4) |
| 2 | 110,379 (20.1) | 2,018 (17.3) |
| 3 | 110,908 (20.2) | 2,278 (19.5) |
| 4 | 109,780 (20.0) | 2,594 (22.2) |
| 5 | 108,944 (19.8) | 2,979 (25.5) |
| ***Assisted Reproductive Technology used*** | 21,004 (3.7) | 441 (3.6) |
| ***Admission status*** |  |  |
| Public | 419,157 (73.3) | 8,582 (70.6) |
| Private | 139,112 (24.3) | 3,276 (26.9) |
| Missing | 13,729 (2.4) | 303 (2.5) |
| ***Previous stillbirth*** | 7,224 (1.3) | 115 (0.9) |
| ***Previous caesarean Section*** | 96,922 (16.9) | 1,829 (15.0) |
| ***Pre-existing diabetes mellitus*** | 4,789 (0.8) | 60 (0.5) |
| ***Pre-existing hypertension*** | 5,890 (1.0) | 103 (0.8) |
| ***Gestational age at first antenatal visit*** | |  |
| <12 weeks | 267,671 (46.8) | 5,302 (43.6) |
| 12-17 weeks | 190,315 (33.3) | 3,911 (32.2) |
| >= 18 weeks | 109,369 (19.1) | 2,801 (23.0) |
| None or inadequately defined | 4,635 (0.8) | 147 (1.2) |
| Missing | 8 (0.0) | 0 |
| ***Smoking status at 20 weeks gestation*** | |  |
| Non-smoker | 495,748 (86.7) |  |
| Quit smoking <20 weeks | 9,125 (1.6) | 166 (1.4) |
| Smoker | 61,011 (10.7) | 684 (5.6) |
| Missing | 6,114 (1.1) | 135 (1.1) |

**Supplementary Table 2.** Pregnancy conditions and labour, birth and perinatal outcomes by maternal region of birth (N=571,988)

| **Characteristics** | **Overall  (N = 571,998)** | **Australia (N = 354,977, 62%)** | **New Zealand  (N = 12,573, 2.2%)** | **Oceania  (N = 3,850, 0.6%)** | **South-East and East Asia  (N = 67,271, 11.8%)** | **South Asia  (N = 64,299, 11.2%)** | **Europe  (N = 26,575, 4.6%)** | **Africa  (N = 18,179, 3.2%)** | **Middle East  (N = 12,113, 2.1%)** | **Other  (N = 12,161, 2.1%)** | **p-value** |
| --- | --- | --- | --- | --- | --- | --- | --- | --- | --- | --- | --- |
| ***Gestational age at first antenatal visit*** |  |  |  |  |  |  |  |  |  |  | <0.001 |
| <12 weeks | 267,671 (46.8) | 180,130 (50.7) | 4,453 (35.4) | 1,115 (29.0) | 29,865 (44.4) | 24,861 (38.7) | 12,094 (45.5) | 5,569 (30.6) | 4,282 (35.4) | 5,302 (43.6) |  |
| 12-17 weeks | 190,315 (33.3) | 112,215 (31.6) | 4,153 (33.0) | 1,169 (30.4) | 23,423 (34.8) | 25,286 (39.3) | 9,414 (35.4) | 6,387 (35.1) | 4,357 (36.0) | 3,911 (32.2) |  |
| >= 18 weeks | 109,369 (19.1) | 59,594 (16.8) | 3,797 (30.2) | 1,498 (38.9) | 13,516 (20.1) | 13,850 (21.5) | 4,891 (18.4) | 6,037 (33.2) | 3,385 (27.9) | 2,801 (23.0) |  |
| None or inadequately defined | 4,635 (0.8) | 3,031 (0.9) | 170 (1.4) | 68 (1.8) | 466 (0.7) | 302 (0.5) | 176 (0.7) | 186 (1.0) | 89 (0.7) | 147 (1.2) |  |
| Missing | 8 (0.0) | 7 (0.0) | 0 (0.0) | 0 (0.0) | <5 | 0 (0.0) | 0 (0.0) | 0 (0.0) | 0 (0.0) | 0 (0.0) |  |
| ***Antenatal care provider*** |  |  |  |  |  |  |  |  |  |  | <0.001 |
| Obstetrician | 305,948 (53.5) | 194,461 (54.8) | 5,858 (46.6) | 2,043 (53.1) | 36,275 (53.9) | 33,068 (51.4) | 13,603 (51.2) | 8,656 (47.6) | 5,976 (49.3) | 6,008 (49.4) |  |
| Midwife | 188,423 (32.9) | 104,432 (29.4) | 5,023 (40.0) | 1,482 (38.5) | 25,456 (37.8) | 24,727 (38.5) | 9,385 (35.3) | 7,961 (43.8) | 5,165 (42.6) | 4,792 (39.4) |  |
| General Practitioner | 73,412 (12.8) | 53,374 (15.0) | 1,505 (12.0) | 243 (6.3) | 5,130 (7.6) | 6,193 (9.6) | 3,444 (13.0) | 1,405 (7.7) | 887 (7.3) | 1,231 (10.1) |  |
| None | 1,537 (0.3) | 998 (0.3) | 107 (0.9) | 49 (1.3) | 149 (0.2) | 52 (0.1) | 39 (0.1) | 64 (0.4) | 17 (0.1) | 62 (0.5) |  |
| Missing | 2,678 (0.5) | 1,712 (0.5) | 80 (0.6) | 33 (0.9) | 261 (0.4) | 259 (0.4) | 104 (0.4) | 93 (0.5) | 68 (0.6) | 68 (0.6) |  |
| ***Gestational diabetes mellitus*** | 62,923 (11.0) | 27,687 (7.8) | 1,142 (9.1) | 709 (18.4) | 11,563 (17.2) | 14,685 (22.8) | 2,145 (8.1) | 2,165 (11.9) | 1,804 (14.9) | 1,023 (8.4) | <0.001 |
| ***Gestational hypertension*** | 14,809 (2.6) | 10,992 (3.1) | 362 (2.9) | 112 (2.9) | 840 (1.2) | 1,103 (1.7) | 637 (2.4) | 316 (1.7) | 169 (1.4) | 278 (2.3) | <0.001 |
| ***Eclampsia*** | 243 (0.0) | 161 (0.0) | 8 (0.1) | <5 | 17 (0.0) | 28 (0.0) | 9 (0.0) | 9 (0.0) | 8 (0.1) | <5 | 0.192 |
| ***Preeclampsia*** | 11,406 (2.0) | 7,833 (2.2) | 302 (2.4) | 139 (3.6) | 836 (1.2) | 1,129 (1.8) | 423 (1.6) | 387 (2.1) | 169 (1.4) | 188 (1.5) | <0.001 |
| ***HELLP Syndrome*** | 694 (0.1) | 447 (0.1) | 20 (0.2) | <5 | 53 (0.1) | 81 (0.1) | 38 (0.1) | 32 (0.2) | 5 (0.0) | 14 (0.1) | 0.002 |
| ***Onset of Labour*** |  |  |  |  |  |  |  |  |  |  | <0.001 |
| Spontaneous | 277,533 (48.5) | 169,070 (47.6) | 6,689 (53.2) | 2,030 (52.7) | 36,828 (54.7) | 27,813 (43.3) | 13,359 (50.3) | 9,160 (50.4) | 6,245 (51.6) | 6,339 (52.1) |  |
| Induced | 174,525 (30.5) | 110,197 (31.0) | 3,773 (30.0) | 1,180 (30.6) | 17,976 (26.7) | 21,445 (33.4) | 7,782 (29.3) | 5,224 (28.7) | 3,502 (28.9) | 3,446 (28.3) |  |
| No labour | 119,854 (21.0) | 75,657 (21.3) | 2,107 (16.8) | 640 (16.6) | 12,457 (18.5) | 15,033 (23.4) | 5,427 (20.4) | 3,793 (20.9) | 2,364 (19.5) | 2,376 (19.5) |  |
| Missing | 86 (0.0) | 53 (0.0) | <5 | 0 (0.0) | 10 (0.0) | 8 (0.0) | 7 (0.0) | <5 | <5 | 0 (0.0) |  |
| ***Iatrogenic birth*** | 294,384 (51.5) | 185,858 (52.4) | 5,880 (46.8) | 1,820 (47.3) | 30,433 (45.2) | 36,478 (56.7) | 13,209 (49.7) | 9,018 (49.6) | 5,866 (48.4) | 5,822 (47.9) | <0.001 |
| ***Mode of Delivery*** |  |  |  |  |  |  |  |  |  |  | <0.001 |
| Vaginal, non-instrumental | 291,550 (51.0) | 183,310 (51.6) | 7,580 (60.3) | 2,475 (64.3) | 34,200 (50.8) | 26,996 (42.0) | 13,373 (50.3) | 10,333 (56.8) | 7,181 (59.3) | 6,102 (50.2) |  |
| Vaginal, instrumental | 88,466 (15.5) | 53,235 (15.0) | 1,572 (12.5) | 307 (8.0) | 11,690 (17.4) | 11,925 (18.5) | 4,592 (17.3) | 1,797 (9.9) | 1,364 (11.3) | 1,984 (16.3) |  |
| Planned CS | 99,318 (17.4) | 63,468 (17.9) | 1,699 (13.5) | 468 (12.2) | 10,791 (16.0) | 11,548 (18.0) | 4,576 (17.2) | 2,901 (16.0) | 1,908 (15.8) | 1,959 (16.1) |  |
| Emergency CS | 92,633 (16.2) | 54,945 (15.5) | 1,721 (13.7) | 600 (15.6) | 10,584 (15.7) | 13,830 (21.5) | 4,034 (15.2) | 3,145 (17.3) | 1,659 (13.7) | 2,115 (17.4) |  |
| Missing | 31 (0.0) | 19 (0.0) | <5 | 0 (0.0) | 6 (0.0) | 0 (0.0) | 0 (0.0) | <5 | <5 | <5 |  |
| **Stillbirth** | 2,434 (0.4) | 1,456 (0.4) | 61 (0.5) | 25 (0.6) | 218 (0.3) | 327 (0.5) | 89 (0.3) | 141 (0.8) | 62 (0.5) | 55 (0.5) | <0.001 |
| ***Timing of stillbirth*** |  |  |  |  |  |  |  |  |  |  | <0.001 |
| Antepartum | 1,931 (79.3) | 1,155 (79.3) | 50 (82.0) | 19 (76.0) | 179 (82.1) | 256 (78.3) | 69 (77.5) | 111 (78.7) | 48 (77.4) | 44 (80.0) |  |
| Intrapartum | 357 (14.7) | 226 (15.5) | 5 (8.2) | <5 | 27 (12.4) | 47 (14.4) | 12 (13.5) | 19 (13.5) | 9 (14.5) | 8 (14.5) |  |
| Unknown timing | 146 (6.0) | 75 (5.2) | 6 (9.8) | <5 | 12 (5.5) | 24 (7.3) | 8 (9.0) | 11 (7.8) | 5 (8.1) | <5 |  |
| ***Gestation of stillbirth*** |  |  |  |  |  |  |  |  |  |  |  |
| Preterm | *1914 (78.6)* | 1152 (79.1) | 43 (70.5) | 19 (76.0) | 106 (75.2) | 48 (77.4) | 260 (79.5) | 169 (77.5) | 71 (79.8) | 46 (83.6) |  |
| Term | 520 (21.3) | 304 (20.9) | 18 (29.5) | 6 (24.0) | 35 (24.8) | 14 (22.6) | 67 (20.5) | 49 (22.5) | 18 (19.2) | 9 (16.4) |  |
| ***Sex of infant*** |  |  |  |  |  |  |  |  |  |  | <0.001 |
| Female | 279,736 (48.9) | 173,909 (49.0) | 6,032 (48.0) | 1,922 (49.9) | 32,543 (48.4) | 31,365 (48.8) | 13,061 (49.1) | 8,943 (49.2) | 5,948 (49.1) | 6,013 (49.4) |  |
| Male | 292,053 (51.1) | 180,952 (51.0) | 6,537 (52.0) | 1,927 (50.1) | 34,707 (51.6) | 32,904 (51.2) | 13,508 (50.8) | 9,220 (50.7) | 6,159 (50.8) | 6,139 (50.5) |  |
| Missing | 209 (0.0) | 116 (0.0) | <5 | <5 | 21 (0.0) | 30 (0.0) | 6 (0.0) | 16 (0.1) | 6 (0.0) | 9 (0.1) |  |
| ***Gestation of birth*** |  |  |  |  |  |  |  |  |  |  | <0.001 |
| Preterm (<37 weeks) | 35,990 (6.3) | 22,953 (6.5) | 742 (5.9) | 272 (7.1) | 3,732 (5.5) | 4,297 (6.7) | 1,408 (5.3) | 1,137 (6.3) | 700 (5.8) | 749 (6.2) |  |
| Term (37-41 weeks) | 532,939 (93.2) | 330,057 (93.0) | 11,749 (93.4) | 3,544 (92.1) | 63,328 (94.1) | 59,765 (92.9) | 24,997 (94.1) | 16,820 (92.5) | 11,380 (93.9) | 11,299 (92.9) | |
| Postterm (>=42 weeks) | 3,069 (0.5) | 1,967 (0.6) | 82 (0.7) | 34 (0.9) | 211 (0.3) | 237 (0.4) | 170 (0.6) | 222 (1.2) | 33 (0.3) | 113 (0.9) |  |
| ***Birthweight below 3rd percentile*** | 11,825 (2.1) | 5,963 (1.7) | 206 (1.6) | 97 (2.5) | 1,609 (2.4) | 2,542 (4.0) | 430 (1.6) | 483 (2.7) | 260 (2.1) | 235 (1.9) | <0.001 |
| ***Special care nursery*** | 75,526 (13.2) | 47,764 (13.5) | 1,549 (12.3) | 597 (15.5) | 7,983 (11.9) | 10,021 (15.6) | 2,932 (11.0) | 2,025 (11.1) | 1,317 (10.9) | 1,338 (11.0) | <0.001 |
| ***Neonatal intensive care unit*** | 6,847 (1.2) | 4,258 (1.2) | 142 (1.1) | 58 (1.5) | 616 (0.9) | 823 (1.3) | 302 (1.1) | 348 (1.9) | 146 (1.2) | 154 (1.3) | <0.001 |
| ***Neonatal death*** | 716 (0.1) | 412 (0.1) | 19 (0.2) | 11 (0.3) | 85 (0.1) | 105 (0.2) | 28 (0.1) | 34 (0.2) | 11 (0.1) | 11 (0.1) | <0.001 |
| ***Perinatal death*** | 3,180 (0.6) | 1,881 (0.5) | 81 (0.6) | 34 (0.9) | 310 (0.5) | 434 (0.7) | 123 (0.5) | 174 (1.0) | 76 (0.6) | 67 (0.6) | <0.001 |

**Supplementary Table 3.** Association between region of birth and stillbirth in Victoria, 2012-2019. All births ≥24+0 weeks gestation (excluding multiple births, congenital anomalies, births to Aboriginal women, and terminations of pregnancy). Odds ratios (with 95% confidence intervals) compared to having a live birth, estimated using logistic regression models (N=570,614)

|  |  | **Minimal Model** | | | | **Model 1** | | |
| --- | --- | --- | --- | --- | --- | --- | --- | --- |
|  | **Stillbirth Rate**  **per 1000 births** | **OR** | **95% C.I.** | | **OR** | | **95% C.I.** | |
| **Overall** | 4.3 |  |  |  |  | |  |  |
|  |  |  |  |  |  | |  |  |
| ***Region of birth*** |  |  |  |  |  | |  |  |
| Australia | 4.1 | 1.00 |  |  | 1.00 | |  |  |
| New Zealand | 4.9 | 1.16 | 0.83 | 1.61 | 1.20 | | 0.86 | 1.69 |
| Oceania | 6.5 | 1.56 | 0.94 | 2.61 | 1.59 | | 0.95 | 2.65 |
| South-East and East Asia | 3.2 | 0.82 | 0.69 | 0.99 | 0.91 | | 0.76 | 1.11 |
| South Asia | 5.1 | 1.21 | 1.03 | 1.41 | 1.16 | | 0.98 | 1.37 |
| Europe | 3.4 | 0.74 | 0.55 | 0.98 | 0.81 | | 0.60 | 1.09 |
| Africa | 7.8 | 1.77 | 1.41 | 2.23 | 1.69 | | 1.33 | 2.16 |
| Middle East | 5.1 | 1.26 | 0.92 | 1.75 | 1.23 | | 0.88 | 1.72 |
| Other | 4.5 | 1.08 | 0.76 | 1.53 | 1.13 | | 0.79 | 1.63 |

^a^ There was no significant interaction between region of birth and the following variables in an unadjusted model: year of birth (p=0.082), maternal age (p=0.204), iatrogenic birth (p=0.401), and preterm birth (p=0.346)

Minimal model = adjusted for year of birth

Model 1 = adjusted for maternal age at birth, primiparity (ref= yes), BMI (ref = ’healthy weight’ 18.5-24.9, IRSD quintiles (continuous), IVF (ref = no), iatrogenic birth (ref = no), smoking status (ref=non-smoker), gestational age at first antenatal care visit (ref = <12 weeks)

**Supplementary Table 4.** Association between region of birth and timing of stillbirth in Victoria, 2012-2019.

All births between ≥20+0 weeks gestation (excluding congenital anomalies, indigenous births, and terminations of pregnancy). Odds ratios (with 95% confidence intervals) estimated compared to having a live birth, using multinominal logistic regression models (N=571,998)

|  | **Stillbirth Rate**  **per 1000 births** | | **Minimal Model** | | | | | | **Model 1** | | | | | |
| --- | --- | --- | --- | --- | --- | --- | --- | --- | --- | --- | --- | --- | --- | --- |
|  | **Antepartum stillbirth** | **Intrapartum stillbirth** | **Antepartum stillbirth** | | | **Intrapartum stillbirth** | | | **Antepartum stillbirth** | | | **Intrapartum stillbirth** | | |
|  |  |  | **OR** | **95%C.I.** | | **OR** | **95%C.I.** | | **OR** | **95%C.I.** | | **OR** | **95%C.I.** | |
| **Region of birth** |  |  |  |  |  |  |  |  |  |  |  |  |  |  |
| Australia | 3.3 | 0.6 | 1.00 |  |  | 1.00 |  |  | 1.00 |  |  | 1.00 |  |  |
| New Zealand | 3.9 | 0.4 | 1.21 | 0.90 | 1.61 | 0.65 | 0.27 | 1.58 | 1.25 | 0.93 | 1.68 | 0.58 | 0.24 | 1.41 |
| Oceania | 4.9 | 1.0 | 1.55 | 0.98 | 2.44 | 1.67 | 0.62 | 4.50 | 1.43 | 0.89 | 2.29 | 1.47 | 0.54 | 3.98 |
| South-East and East Asia | 2.7 | 0.4 | 0.83 | 0.71 | 0.97 | 0.64 | 0.42 | 0.95 | 0.92 | 0.77 | 1.08 | 0.61 | 0.39 | 0.95 |
| South Asia | 3.9 | 0.7 | 1.27 | 1.10 | 1.45 | 1.22 | 0.89 | 1.68 | 1.23 | 1.07 | 1.42 | 1.25 | 0.89 | 1.76 |
| Europe | 2.6 | 0.5 | 0.81 | 0.64 | 1.04 | 0.73 | 0.41 | 1.29 | 0.87 | 0.68 | 1.12 | 0.80 | 0.45 | 1.44 |
| Africa | 6.1 | 1.1 | 1.91 | 1.57 | 2.32 | 1.71 | 1.07 | 2.74 | 1.76 | 1.43 | 2.16 | 1.51 | 0.92 | 2.48 |
| Middle East | 3.9 | 0.5 | 1.25 | 0.94 | 1.67 | 1.22 | 0.62 | 2.37 | 1.21 | 0.90 | 1.63 | 0.99 | 0.48 | 2.02 |
| Other | 3.6 | 0.7 | 1.10 | 0.81 | 1.49 | 1.04 | 0.51 | 2.11 | 1.16 | 0.85 | 1.59 | 1.10 | 0.54 | 2.24 |

Minimal model = adjusted for year of birth

Model 1 = adjusted for maternal age at birth, primiparity (ref= yes), BMI (ref = ’healthy weight’ 18.5-24.9, IRSD quintile (continuous), IVF (ref = no), iatrogenic birth (ref = no), smoking status (ref=non-smoker), gestational age at first antenatal care visit (ref = <12 weeks)

**Supplementary Table 5.** Stillbirth rate by cause (PSANZ perinatal death classification) and region, among singleton births ≥20 weeks gestation (including congenital anomalies and terminations of pregnancy, but excluding multiple births, and births to Aboriginal and/or Torres Strait Islander women) (N=603,081)

|  |  | **Stillbirth Rate by Cause per 1000 Births per Region** | | | | | | | | |
| --- | --- | --- | --- | --- | --- | --- | --- | --- | --- | --- |
| **Characteristics** | **Total stillbirths, N=4,893** | **Australia (N = 375,264, 62.2%)** | **New Zealand  (N = 13,211, 2.2%)** | **Oceania  (N = 4,019, 0.7%)** | **South-East and East Asia  (N = 70,643, 11.7%)** | **South Asia  (N = 67,225, 11.2%)** | **Europe  (N = 28,040, 4.7%)** | **Africa  (N = 19,119, 3.2%)** | **Middle East  (N = 12,759, 2.1%)** | **Other  (N = 12,801, 2.1%)** |
|  |  |  |  |  |  |  |  |  |  |  |
| ***Perinatal death classification*** |  |  |  |  |  |  |  |  |  |  |
| Congenital anomaly | 1,415 | 2.33 | 1.74 | 2.98 | 1.94 | 2.73 | 2.10 | 2.71 | 2.35 | 3.12 |
| Maternal conditions | 1,131 | 1.79 | 5.29 | 7.71 | 1.37 | 1.87 | 1.78 | 1.77 | 1.18 | 2.97 |
| Unexplained antepartum fetal death | 783 | 1.29 | 2.04 | 1.24 | 0.94 | 1.33 | 1.18 | 1.98 | 1.96 | 0.94 |
| Placental dysfunction or causative placental pathology | 399 | 0.61 | 0.83 | 1.24 | 0.42 | 1.10 | 0.46 | 1.05 | 0.71 | 0.62 |
| Spontaneous preterm labour or rupture of membranes (<37 weeks) | 341 | 0.50 | 0.45 | 0.25 | 0.52 | 0.74 | 0.43 | 1.46 | 0.78 | 0.70 |
| Special perinatal conditions | 282 | 0.46 | 0.45 | 0.75 | 0.47 | 0.39 | 0.43 | 0.58 | 0.63 | 0.70 |
| Antepartum haemorrhage | 232 | 0.39 | 0.23 | 0.49 | 0.27 | 0.49 | 0.14 | 0.78 | 0.24 | 0.47 |
| Perinatal infection | 131 | 0.23 | 0.23 | 0.25 | 0.11 | 0.25 | 0.14 | 0.31 | 0.31 | 0.23 |
| Hypertension | 86 | 0.14 | 0 | 0.49 | 0.06 | 0.22 | 0.11 | 0.31 | 0.16 | 0.16 |
| Hypoxic peripartum death | 48 | 0.10 | 0 | 0 | 0.04 | 0.06 | 0.03 | 0.05 | 0 | 0 |
| Missing | 45 | 0.06 | 0 | 0.49 | 0.07 | 0.07 | 0.04 | 0.31 | 0.08 | 0.08 |


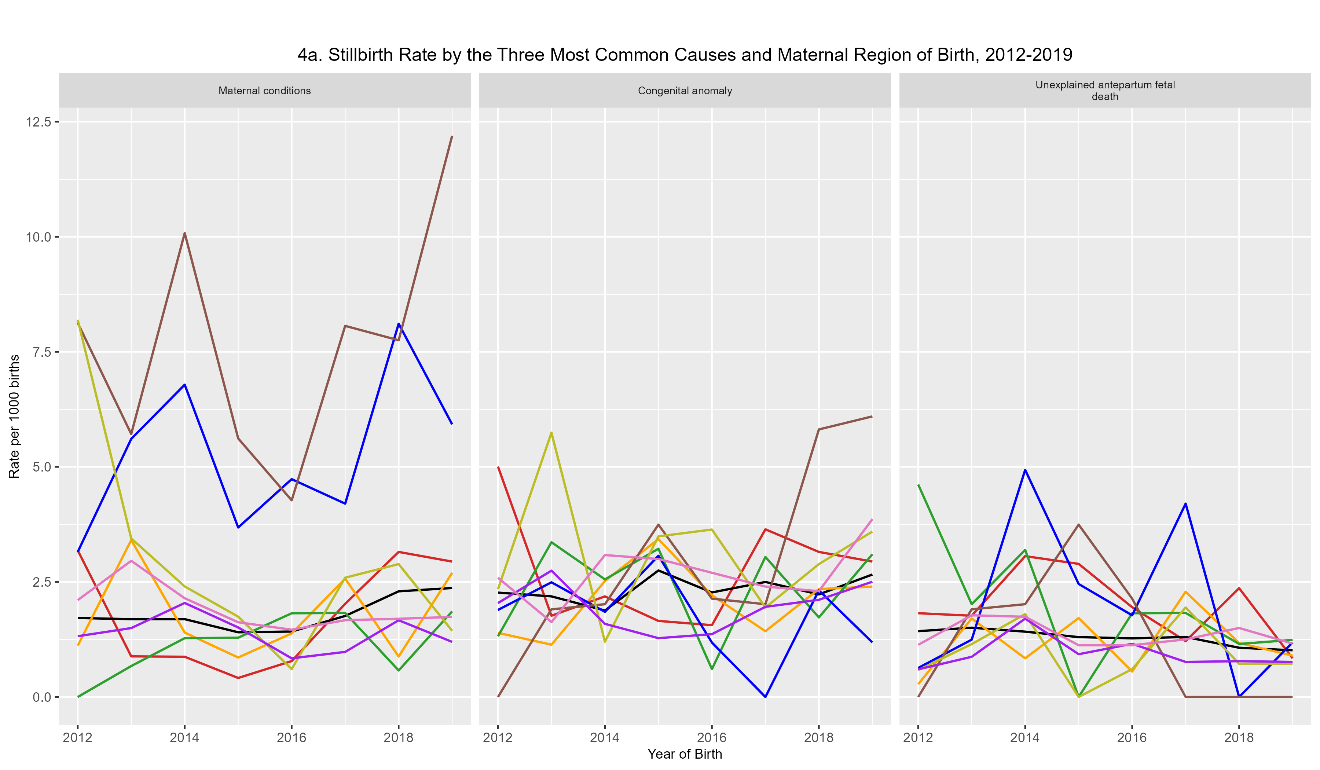

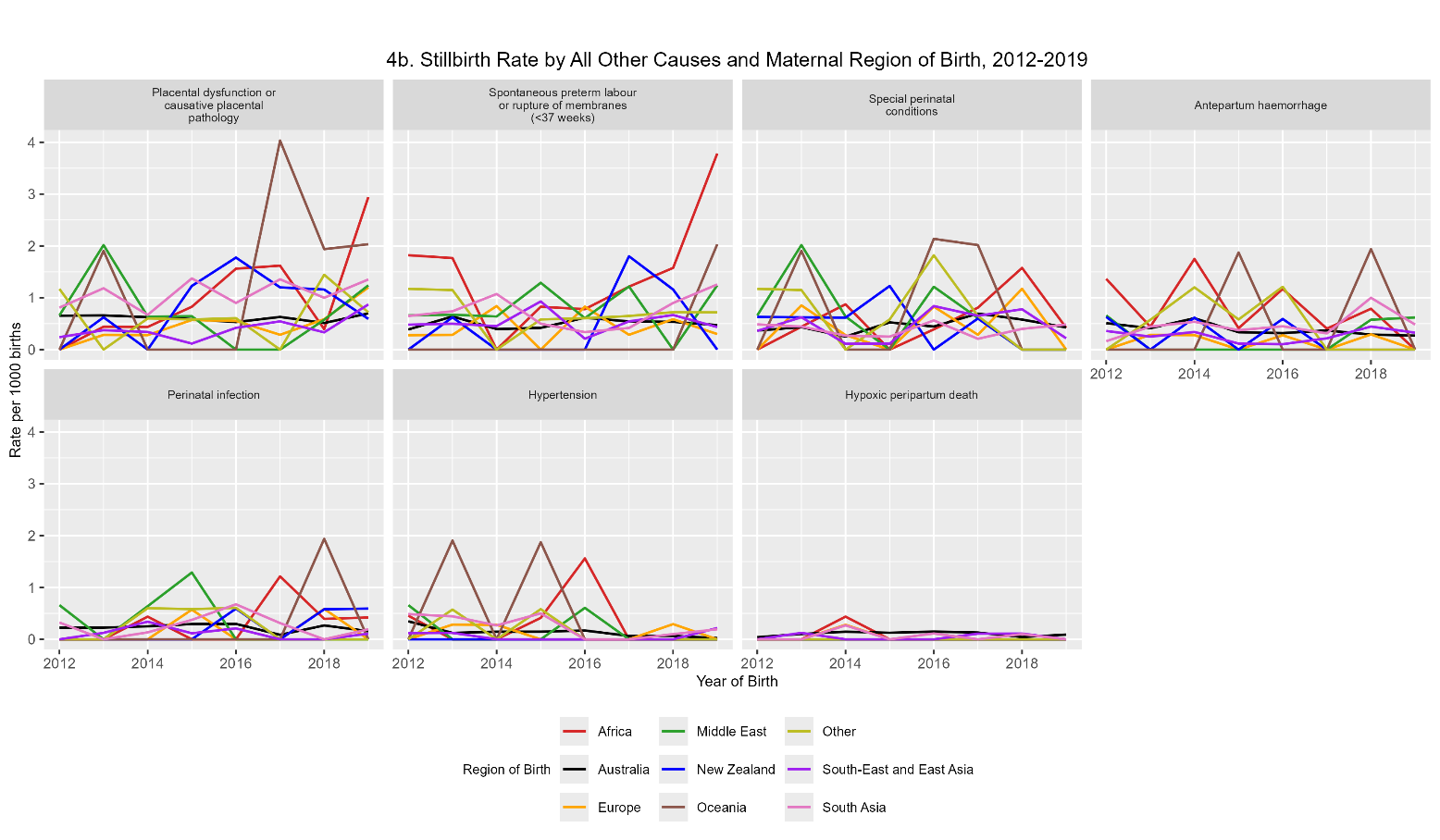


**Supplementary Figure 1.** Trend in stillbirth rate by maternal region of birth, panelled by a) the three most common causes of stillbirth and b) all other causes
